# Activation of Dorsomedial Hypothalamic Neurons Promotes Physical Activity and Decreases Food Intake and Body Weight in Zucker Fatty Rats

**DOI:** 10.3389/fnmol.2018.00179

**Published:** 2018-05-29

**Authors:** Ni Zhang, Liang Yang, Lanting Guo, Sheng Bi

**Affiliations:** ^1^Department of Psychiatry and Behavioral Sciences, Johns Hopkins University School of Medicine, Baltimore, MD, United States; ^2^Department of Psychiatry, West China Hospital, Sichuan University, Chengdu, China

**Keywords:** physical activity, running wheel, food intake, body weight, obesity, leptin, leptin receptor, dorsomedial hypothalamus

## Abstract

Previous reports have shown that running wheel activity or voluntary exercise prevents hyperphagia and obesity in various animal models of obesity, but such effects seem only minimal in obese animals lacking leptin or leptin receptors. The mechanisms underlying this ineffectiveness remain unclear. Here, we identified the action of neuronal activation in the dorsomedial hypothalamus (DMH) in modulating physical activity, food intake and body weight using leptin receptor mutant obese Zucker (*Lepr(fa)*, ZF) and Koletsky (*Lepr(fak)*, SHROB) rats. Ad lib-fed SHROB rats with locked running wheels became hyperphagic and gained body weight rapidly. These alterations were not ameliorated in ad lib-fed SHROB rats with voluntary access to running wheels, but the body weight of SHROB rats with running wheel access was significantly decreased when they were pair-fed to the amounts consumed by lean controls. Determinations of hypothalamic gene expression revealed that sedentary ad lib-fed SHROB rats had increased expression of neuropeptide Y (*Npy*) and decreased expression of pro-opiomelanocortin (*Pomc*) in the arcuate nucleus (ARC). Both ARC *Npy* and *Pomc* expression were further altered under running and pair-fed conditions, indicating that both genes are appropriately regulated in response to increased energy demands or alterations caused by running activity and food restriction. Strikingly, c-Fos immunohistochemistry revealed that while voluntary running activity elevated the number of c-Fos positive cells in the DMH (particularly in the ventral and caudal subregions) of intact rats, such activation was not observed in ZF rats. Using adeno-associated virus (AAV)-mediated expression of the designer receptors hM3D(Gq) in the ventral and caudal DMH of ZF rats, we found that chemogenetic stimulation of neurons in these DMH subregions via injection of the designer drug clozapine N-oxide (CNO) significantly increased their running activity and reduced their food intake and body weight. Together, these results demonstrate that activation of ventral and caudal DMH neurons promotes physical activity and decreases food intake and body weight and suggest that intact DMH neural signaling is likely crucial for exercise-induced reductions of food intake and body weight in obese rats lacking leptin receptors.

## Introduction

Obesity has become a public health problem and has been linked to various life-threatening diseases such as cardiovascular disease and type 2 diabetes. Regular physical exercise provides numerous health benefits and is one of the most important strategies for preventing obesity and lowering the risk of obesity-associated comorbidities. Notably, physical exercise improves weight loss and, when added to dietary control, becomes a key factor for success in long-term weight maintenance in previously overweight and obese individuals (Wing and Hill, 2001). Intense exercise reduces daily energy balance in adolescents with obesity by both increasing energy expenditure and decreasing food consumption (Thivel et al., 2013). Using rodent obesity models, we and other investigators have demonstrated an important role for physical exercise in the prevention or control of obesity. Voluntary access to running wheels or running activity ameliorates hyperphagia and obesity of Otsuka Long-Evans Tokushima Fatty (OLETF) rats lacking cholecystokinin (CCK) 1 receptors (Bi et al., 2005); chronic exercise lowers the defended body weight and adiposity in diet-induced obese (DIO) rats (Levin and Dunn-Meynell, 2004); and voluntary exercise delays monogenetic obesity and overcomes reproductive dysfunction of melanocortin-4 receptor (MC4R) knockout mice (Irani et al., 2005). In contrast, physical exercise appears less effective in obese animals with a deficit in leptin signaling. Voluntary exercise has only minimal or no effect on obesity in leptin deficient *ob/ob* mice (Dubuc et al., 1984) or leptin receptor deficient Zucker fatty rats (Stern and Johnson, 1977). Thus, while physical exercise exerts a profound action in the control of obesity in some obesity models, it is unclear why physical exercise is ineffective in obese animals lacking leptin or leptin receptors.

Leptin, a hormone produced in adipose tissue in mammals, acts as an adiposity feedback signal to affect brain’s control of energy homeostasis (Cone, 2006; Morton et al., 2006; Gautron and Elmquist, 2011; Friedman, 2014). The hypothalamus plays a fundamental role in maintaining energy balance via regulating food intake and energy expenditure; leptin modulates these hypothalamic actions. Particularly, the arcuate nucleus (ARC) of the hypothalamus contains two populations of leptin receptor-containing neurons: the orexigenic neurons releasing neuropeptide Y (NPY) and the endogenous melanocortin receptor antagonist agouti-related protein (AgRP; Mercer et al., 1996; Hahn et al., 1998) and the anorectic proopiomelanocortin (POMC) neurons producing α-melanocyte-stimulating hormone (α-MSH; Cheung et al., 1997). Leptin inhibits NPY/AgRP neurons and activates POMC neurons (Morton et al., 2006; Gautron and Elmquist, 2011). Thus, elevation of leptin signaling causes decreased food intake and body weight loss, whereas decreased leptin signaling produces opposite effects (Morton et al., 2006; Gautron and Elmquist, 2011; Friedman, 2014). Based on this notion, one might expect that physical exercise promotes leptin’s actions, leading to subsequent inhibitory effects on food intake and body weight. Paradoxically, Zachwieja et al. (1997) found that voluntary running exercise decreases adipose leptin mRNA and circulating leptin levels in both obese and lean rats. We have demonstrated that voluntary running activity normalizes food intake, body weight, and plasma glucose levels, as well as prevents elevated plasma leptin levels in OLETF rats (Bi et al., 2005). Intriguingly, others have reported that postweaning exercise produces prolonged increases in central leptin sensitivity and signaling in DIO rats (Patterson et al., 2009). Despite these observations, how hypothalamic leptin signaling contributes to the feeding and body weight effects of physical exercise remains unclear.

Zucker fatty (*fa*/*fa*, ZF) and spontaneously hypertensive obese Koletsky (*fa^k^*/*fa^k^*, SHROB) rats are animal models of obesity, and both strains have mutant leptin receptor in the fatty allele (*Lepr(fa)* and *Lepr(fak)* respectively), resulting in leptin signaling deficiency (Chua et al., 1996; Phillips et al., 1996; Takaya et al., 1996; Wu-Peng et al., 1997). In addition to the vascular disease phenotype in SHROB rats, both ZF and SHROB rats develop hyperphagia, obesity, hyperinsulinemia and hyperlipidemia (Argilés, 1989; Ernsberger et al., 1999). In the present study, we sought to use these rat models to evaluate the potential role for leptin signaling in exercise-induced reductions of food intake and body weight. Animals had voluntary access to running wheels, and we examined the effects of running activity on food intake, body weight, and ARC *Npy* and *Pomc* mRNA expression. Since exercise affects mRNA expression of *Npy* and anorectic corticotropin-releasing factor (*Crf*) in the dorsomedial hypothalamus (DMH) of OLETF rats (Bi et al., 2005) and centrally administered leptin increases *Crf* mRNA expression in the paraventricular nucleus (PVN) of intact rats but not Zucker fatty rats (Schwartz et al., 1996), we also examined the effects of running activity on the expression of these genes. Furthermore, we used the immediate early gene product c-Fos as a marker of neuronal activation to explore exercise-induced neuronal activation in the brain. The designer receptors exclusively activated by designer drugs (DREADD)-based chemogenetic tools provide unique approaches for the *in vivo* study of neuronal functions (Roth, 2016). Among G protein-coupled DREADDs, the hM3D(Gq) DREADD contains engineered human M3 muscarinic receptors and is typically used for neuronal excitation (Roth, 2016). This receptor can be activated by the selective designer drug clozapine N-oxide (CNO; Armbruster et al., 2007). Finally, we used the hM3D(Gq) DREADD to further determine a role for DMH neuronal activation in modulating physical activity, food intake and body weight.

## Materials and Methods

### Animals

Male obese Koletsky (*Lepr(fak)*, SHROB) rats and age-matched male lean control (CTL) rats were purchased from Charles River Laboratories, Inc., and were maintained individually in locked running wheel cages on a 12:12 h dark/light cycle (lights on at 06:00h) in a temperature-controlled colony room (22–24°C). All rats had ad lib access to 45-mg food pellets except where noted and water was always available. All procedures were approved by the Institutional Animal Care and Use Committee at the Johns Hopkins University and were in accordance with the National Institute of Health’s Guide for the Care and Use of Laboratory Animals.

### Running Wheel Activity and Pair Feeding

At the beginning of the experiment, four lean rats were maintained in locked running wheel cages with *ad libitum* access to food and served as a sedentary and normal intake control group (CTL-SED). Eight SHROB rats were divided into two groups (*n* = 4): one sedentary group with locked running wheels (SHROB-SED) and the other with voluntary access to running wheels (SHROB-RW). Both groups of SHROB-SED and SHROB-RW rats had *ad libitum* access to food. Running activity and food intake were computer monitored for 24 h/days. Body weight was examined daily. Five weeks after access to running wheels, SHROB-RW rats were pair-fed to the amount and pattern of food that was consumed by CTL-SED rats, i.e., food intake of each pair fed animal was determined by a computerized feeding system that presented food pellets in the same amount and pattern as its “yoked” CTL-SED rat. As previously reported, we used this pair-feeding approach to distinguish feeding or energy expenditure effect on body weight loss in intact rats with voluntary access to running wheels (Zheng et al., 2016). Four weeks later, all rats were sacrificed between 09:00 and 11:00 h by decapitation under isoflurane inhalation anesthesia, and brains were removed and rapidly frozen in isopentane on dry ice for subsequent analyses of hypothalamic gene expression.

### Real Time RT-PCR

Real-time reverse transcription polymerase chain reaction (RT-PCR) was performed for determinations of hypothalamic gene expression in SHROB rats (*n* = 4 per group). Briefly, brains were sectioned via a cryostat at −20°C, three hypothalamic nuclei at the levels of the ARC, the DMH and the PVN were punched out, and saved in RNAlater solutions. Total RNA was extracted from each sample using Trizol reagent (Life Technologies, Grand Island, NY, USA). Quantitative real time RT-PCR was conducted to determine levels of mRNA expression for *Npy*, *Pomc* and *Crf* by using the iScript one-step RT-PCR kit with SYBR Green (Bio-Rad Laboratories, Hercules, CA, USA) on iQ5 Multicolor Real-Time PCR Detection System (Bio-Rad Laboratories, Hercules, CA, USA). β-actin was used as an internal control for quantification of individual mRNA. The formula 2^−ΔΔCT^ was used to quantify relative mRNA expression levels. The first ΔCT was calculated by comparing the Ct (threshold cycle) value of a target gene to an internal control in the sample. The second ΔΔCT was the difference in ΔCT between the treated and control samples. The final value of 2^−ΔΔCT^ was determined as the amount of target gene expression relative to the control group. A list of primer sets included: *Npy* forward primer, 5’-agagatccagccctgagaca-3’, and reverse primer, 5’-aacgacaacaagggaaatgg-3’; *Pomc*, forward primer, 5’-tccatagacgtgtggagctg-3’ and reverse primer, 5’-acgtacttccggggattttc-3’; *Crf*, forward primer, 5’-agaagagagcgcccctaaac-3’ and reverse primer, 5’-atcagaatcggctgaggttg-3’; and β-actin, forward primer, 5’-tgtcaccaactgggacgata-3’ and reverse primer, 5’-ggatggctacgtacatggct-3’.

### c-Fos Immunohistochemistry

One additional cohort of 12 male Zucker fatty (*Lepr(fa)*, ZF) rats was purchased from Charles River Laboratories, Incorporation (ZF rats were purchased because SHROB rats were not supplied) and maintained individually in locked running wheel cages with *ad libitum* access to water and standard rodent chow. After 1 week habituation, seven rats had voluntary access to running wheels (ZF-RW) and five sedentary rats remained in locked running wheels (ZF-SED). For the sake of economy, 10 age-matched male Sprague-Dawley rats served as lean control; half of them had voluntary access to running wheels (LEAN-RW) and the other half remained in locked running wheels as a sedentary control (LEAN-SED). To prevent feeding effect on c-Fos activation determination, chow was removed from the cages 2 h before running wheels were unlocked. Three and a half hours after running wheel access, at a time when running wheel activity promoted strong c-Fos activation based on our pilot study, rats were anesthetized with Euthasol (pentobarbital sodium and phenytoin, Delmarva Laboratories, Midlothian, VA, USA) and perfused transcardially with phosphate-buffered saline (PBS, pH 7.4) followed by 4% paraformaldehyde in PBS as previously described (Chen et al., 2008). Brains were removed and stored in 4% paraformaldehyde at 4°C for 6 h, and were then transferred to 25% sucrose for 48 h at 4°C for subsequent c-Fos immunoreactivity determinations.

Brains from four groups of the rats (5 LEAN-SED, 5 LEAN-RW, 5 ZF-SED and 7 ZF-RW) were processed for c-Fos immunohistochemistry. As previously described (Chen et al., 2008), 40 μm coronal sections through the whole brain region were cut via a cryostat and saved in three series (one in three throughout the brain). A series of sections was processed with our standard c-Fos immunohistochemistry (Chen et al., 2008). c-Fos positive cells were identified using Vectastain Elite ABC kit (Vector Laboratories, Burlingame, CA, USA). Briefly, sections were incubated in 0.3% hydrogen peroxide for 60 min, rinsed in PBS and preabsorbed with normal goat blocking serum in PBS containing 0.1% Triton X-100 for 30 min. They were then incubated in rabbit anti-c-Fos antiserum (1:20,000, Oncogene Science, San Diego, CA, USA) over two nights at room temperature. After six washes in PBS, sections were incubated in biotinylated goat anti-rabbit IgG for 60 min, washed three times in PBS, followed by incubation in ABC reagent (avidin DH:biotinylated horseradish peroxidase H complex) for 30 min. Finally, sections were stained by incubation with 3,3’-diaminobenzidine (DAB) chromagen and nickel enhancer (DAB substrate kit for peroxidase, Vector Laboratories). They were then mounted onto gelatin-coated slides, air dried, dehydrated in an ascending series of ethanol and coverslipped.

The number of c-Fos positive cells was quantified in the following areas: the PVN, the DMH, the ARC, the ventromedial hypothalamus (VMH), the lateral hypothalamus (LH), the area postrema (AP) and the nucleus of the solitary tract (NTS) as well as the central nucleus of amygdala (CeA) according to rat brain atlas (Paxinos and Watson, 2005). Images of sections were captured by digital camera (Retiga 2000R, QImaging, Burnaby, Canada) attached to Zeiss Axio Imager (Carl Zeiss MicroImaging, Inc., Thornwood, NY, USA). The area of interest was outlined based on cellular morphology and c-Fos positive cells were counted by the imaging program (ImageJ, National Institutes of Health, USA). Data for c-Fos activation in all areas were bilaterally assessed and were presented as the total number of c-Fos positive cells per section.

### Chemogenetic Stimulation of DMH Neurons Via DREADD

A subset of 12 male obese ZF rats at 5 weeks of age were purchased and individually housed in locked running wheel cages with *ad libitum* access to water and standard rodent chow. Based on the results of exercise-induced c-Fos activation in the brain, the vector of adeno-associated virus (AAV)-mediated DREADD AAV5-hSyn-hM3D(Gq)-mCherry (hM3D, 5.3 × 10^12^ vg/ml, the University of North Carolina at Chapel Hill, Chapel Hill, NC, USA) was applied for stimulation of neurons in the DMH, primarily in the ventral and caudal subregions. At 6 weeks of age, rats were randomly assigned into to two groups: eight rats received bilateral DMH injections of hM3D and four rats received bilateral DMH injections of the empty vector control (CTL). Briefly, 0.2 μl/site (~1 × 10^9^ particles/site) of the vector hM3D or CTL were injected into the DMH bilaterally with the following coordinates: 2.9 mm caudal to bregma, 0.4 mm lateral to midline and 8.7 mm ventral to skull surface (Paxinos and Watson, 2005; Chao et al., 2011). Each DMH injection was made with a glass micropipette (10–30 microns in tip size) via the Drummond Nanoject II Auto-Nanoliter Injector (Drummond scientific company, Broomall, PA, USA) and the injector remained in place for additional 5 min before removal. Following DMH injection, rats were returned to locked running wheel cages for postoperative recovery, were habituated to the intraperitoneal (ip) injection, and their body weights were measured daily.

At 3 weeks following DMH injection, a feeding test was carried out to determine the effect of neuronal activation in the DMH on food intake. Food was removed from the cages 2 h before lights off. At 30 min before lights off, rats received ip saline or the designer drug CNO (1 mg/kg body weight, Tocris, Minneapolis, MN, USA). The dose of CNO for selectively activating DMH neurons via the hM3D(Gq) was based on previous reports (Armbruster et al., 2007; Roth, 2016) and our pilot study. Food was returned to the cages at lights off (30 min after ip injection) and food intake was measured at 2, 4 and 24 h from lights off. After 3-day recovery, rats received a second injection in a counterbalanced design. Likewise, 2, 4 and 24 h of food intake were determined in animals. After this feeding test (at 4 weeks following DMH injection), both hM3D rats and CTL rats received ip injection of CND (1 mg/kg body weight) daily. IP injection was made at 30 min before lights off. Running wheels were unlocked in both groups after the first injection. Running activity, food intake and body weight were recorded daily for 3 days. On day 4, rats received last injection and 3.5 h later, rats were perfused transcardially with 4% paraformaldehyde as described above, and brains were saved for subsequent verification of DMH DREADD injection. Briefly, 40 μm coronal sections throughout the hypothalamic region were cut via a cryostat and the sections with m-Cherry (red) fluorescence were examined on a Zeiss Axio Imager (Carl Zeiss MicroImaging). Data from 2 hM3D rats with incorrect injection were excluded from subsequent statistical analyses.

### Statistical Analysis

All values are presented as means ± SEM. Data were analyzed by StatSoft Statistica-7 software. Data for body weight and food intake were analyzed using two-way repeated measures ANOVA (analysis of variance). Data for gene expression were analyzed using two-tailed Student *t*-test. Data for c-Fos positive cells were analyzed using two-tailed Student *t*-test. All ANOVA’s were followed by pairwise multiple Fisher’s LSD comparisons. *P* < 0.05 was considered as a statistically significant difference.

## Results

### Effects of Running Wheel Activity on Food Intake and Body Weight

**Figure 1:**
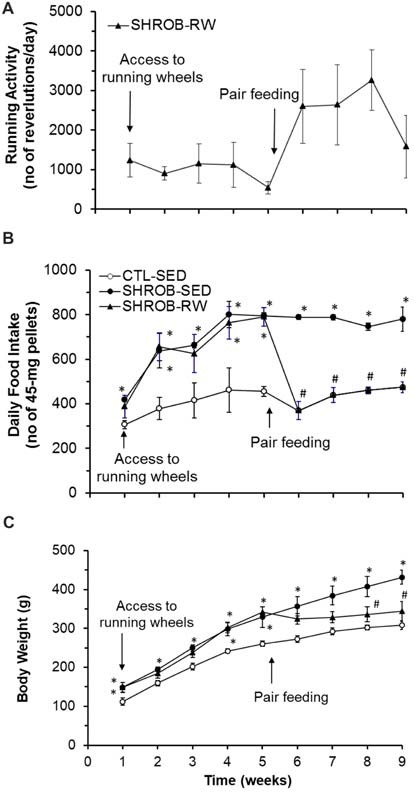
Effects of running wheel activity on food intake and body weight in SHROB rats. **(A)** Running wheel activity in SHROB rats with access to running wheels (SHROB-RW). **(B)** Food intake in lean sedentary control rats with locked running wheels (CTL-SED), sedentary SHROB rats with locked running wheels (SHROB-SED) and SHROB-RW rats. **(C)** Body weight in the three groups of rats. Values are means ± SEM. *n* = 4 rats per group. *P < 0.05 vs. CTL-SED and ^#^*P* < 0.05 vs. SHROB-SED.

During the initial 5-week period of ad lib feeding, SHROB rats with access to running wheels (SHROB-RW) run an average of 500–1200 revolutions daily (about 0.5–1.2 km/day) with the mean of 992 ± 219 revolutions/day (Figure 1A). However, this running activity did not affect food intake. As shown in Figure 1B, food intake was significantly increased in sedentary SHROB rats (SHROB-SED) by about 70% more than lean sedentary control rats (CTL-SED) over the 5 weeks (*F*_(1,6)_ = 119.59, *P* = 0.00003), whereas SHROB-RW rats consumed the same amount of food daily as SHROB-SED rats (*F*_(1,6)_ = 0.199, *P* = 0.671). Thus, SHROB-SED rats gained body weight rapidly compared to CTL-SED rats (*F*_(1,6)_ = 21.006, *P* = 0.004, Figure 1C). Although SHROB-RW rats gained slightly less body weight in weeks 2 and 3 relative to SHROB-SED rats, the degree of difference in body weight was very small (about 5% less than SHROB-SED rats) and the body weights of SHROB-SED and SHROB-RW rats did not significantly differ (*F*_(1,6)_ = 0.006, *P* = 0.942, Figure 1C). As a result, both groups of SHROB rats became significantly heavier, gaining about 30% more weight than CTL-SED rats by week 5 (Figure 1C). These data indicate that voluntary running activity was ineffective in preventing hyperphagia and obesity in SHROB rats, suggesting that SHROB rats appear to have impaired feeding and body weight regulation in response to physical activity.

To assess this impairment, we exploited a food restriction strategy to investigate the feeding effect on body weight gain in SHROB rats. Following 5 weeks of ad lib feeding, SHROB-RW rats were switched to a pair-feeding regimen in which they received food pellets in the same amount and pattern as their “yoked” CTL-SED rats via a computerized feeding system (Figure 1B). Pair feeding promoted running activity in SHROB-RW rats. The running activity of pair-fed SHROB-RW rats was greatly increased from an average of 500–1200 revolutions/day in initial 5 weeks to 1600–3300 revolutions daily (1.6–3.3 km/day) in the four subsequent weeks with the mean of 2331 ± 425 revolutions/day (*P* = 0.049 vs. 992 ± 219 revolutions/day in initial 5 weeks, Figure 1A). As expected, pair-fed SHROB-RW rats consumed the same amount of food as their “yoked” CTL-SED rats (Figure 1B). From week 5 to week 9, SHROB-SED rats remained hyperphagic and continued to gain more body weight (about 103 g), leading to 40% increase in body weight at week 9 compared to CTL-SED rats that gained only 48 g (Figure 1C). Conversely, pair-fed SHROB-RW rats lost about 2 g of body weight in 4 weeks (Figure 1C). As such, the body weight of pair-fed SHROB-RW rats was significantly decreased, being 26% lighter than that of SHROB-SED rats at week 9 (*P* = 0.009) and down to the level that was no longer significantly different from that of CTL-SED rats (*P* = 0.208, Figure 1C). Thus, these data provide evidence that food restriction together with the resulting elevation of running activity prevented body weight gain in SHROB rats, but the specific portion of weight loss in SHROB-RW rats resulting from food restriction alone was unclear.

### Dysregulation of Hypothalamic Gene Expression in Rats With Leptin Receptor Deficiency

**Figure 2:**
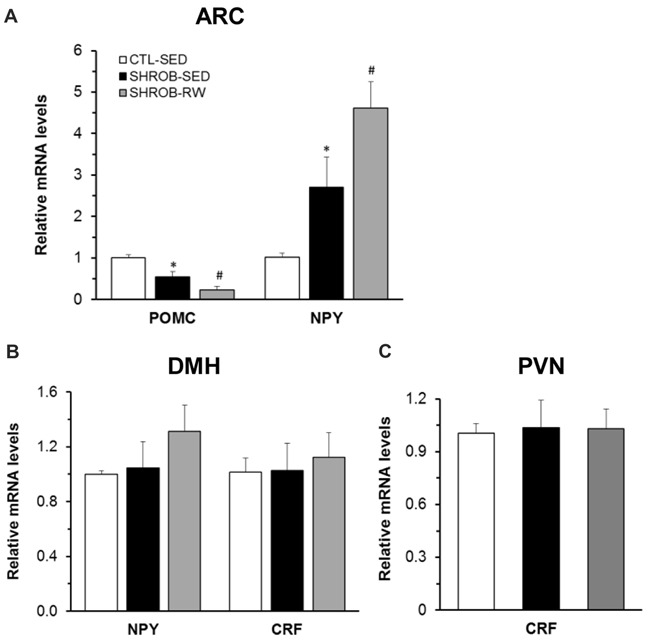
Hypothalamic mRNA expression in SHROB rats. **(A)** Proopiomelanocortin (*Pomc*) and neuropeptide Y (*Npy*) mRNA expression in the arcuate nucleus (ARC). **(B)** *Npy* and corticotrophin-releasing factor (*Crf*) mRNA expression in the dorsomedial hypothalamus (DMH). **(C)** *Crf* mRNA expression in the paraventricular nucleus (PVN). Values are means ± SEM. *n* = 4 rats per group. *P < 0.05 vs. CTL-SED and ^#^*P* < 0.05 vs. SHROB-SED.

At week 9, we evaluated hypothalamic gene expression in SHROB rats. Consistent with loss of functional leptin receptors in SHROB rats (Wu-Peng et al., 1997), SHROB-SED rats had a 54% reduction of *Pomc* mRNA expression (*P* = 0.006) and a 2.7 fold increase in *Npy* mRNA expression in the ARC compared to CTL-SED rats (*P* = 0.046, Figure 2A). These alterations became even larger in pair-fed SHROB-RW rats. Levels of *Pomc* mRNA expression in the ARC of pair-fed SHROB-RW rats were down to 43% of those in SHROB-SED rats (*P* = 0.04, Figure 2A). ARC expression of *Npy* mRNA in pair-fed SHROB-RW rats was increased by 1.7 fold of SHROB-SED rats (*P* = 0.031, Figure 2A). Thus, both ARC *Pomc* and *Npy* expression remain appropriately regulated in response to increased energy demands derived from physical exercise and food restriction despite leptin signaling deficiency.

Within the DMH, both *Npy* and *Crf* gene expression are regulated in response to running wheel access in lean intact and obese OLETF rats (Bi et al., 2005; Kawaguchi et al., 2005). We next assessed whether such regulation is altered in SHROB rats with leptin signaling deficiency. In contrast to ARC NPY, a lack of leptin receptors did not affect *Npy* mRNA expression in the DMH of SHROB-SED (Figure 2B). Levels of DMH *Npy* expression did not differ between the two groups of SHROB-SED rats and CTL-SED rats (Figure 2B). There was a 26% increase in *Npy* mRNA expression in the DMH of pair-fed SHROB-RW rats compared to SHROB-SED rats, but this increase did not reach a statistical significance (*P* = 0.374, Figure 2B). Likewise, *Crf* mRNA expression in the DMH was not significantly altered in SHROB-SED or pair-fed SHROB-RW rats compared to CTL-SED or SHROB-SED rats, respectively (Figure 2B).

Since centrally administered leptin increases *Crf* mRNA expression in the PVN of fasting rats (Schwartz et al., 1996), we examined *Crf* mRNA expression in the PVN of SHROB rats. The expression levels of *Crf* mRNA in the PVN did not significantly differ among the three groups of rats, indicating that neither leptin signaling deficiency nor running activity and food restriction alters PVN *Crf* gene expression (Figure 2C).

### Alterations in Exercise-Induced c-Fos Activation in Rats With Leptin Receptor Deficiency

**Figure 3:**
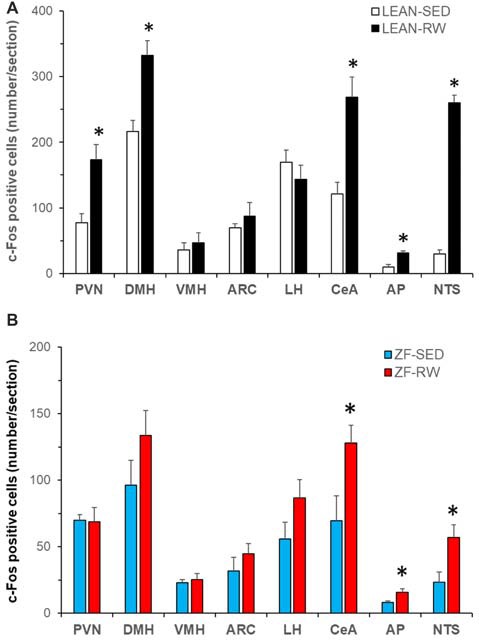
Characterization of running activity-induced brain c-Fos activation in Zucker fatty (ZF) rats. **(A)** Brain c-Fos activation in lean intact rats with running wheel access (LEAN-RW, *n* = 5) compared to lean sedentary control rats with locked running wheels (LEAN-SED, *n* = 5). **(B)** Brain c-Fos activation in ZF rats with running wheel access (ZF-RW, *n* = 7) compared to sedentary ZF rats with locked running wheels (ZF-SED, *n* = 5). VMH: the ventromedial hypothalamus (VMH); LH: the lateral hypothalamus; CeA: the central nucleus of amygdala; AP: area postrema; NTS: the nucleus of the solitary tract. Values are means ± SEM. *n* = 5–7 rats per group. *P < 0.05 vs. sedentary controls (LEAN-SED or ZF-SED).

**Figure 4:**
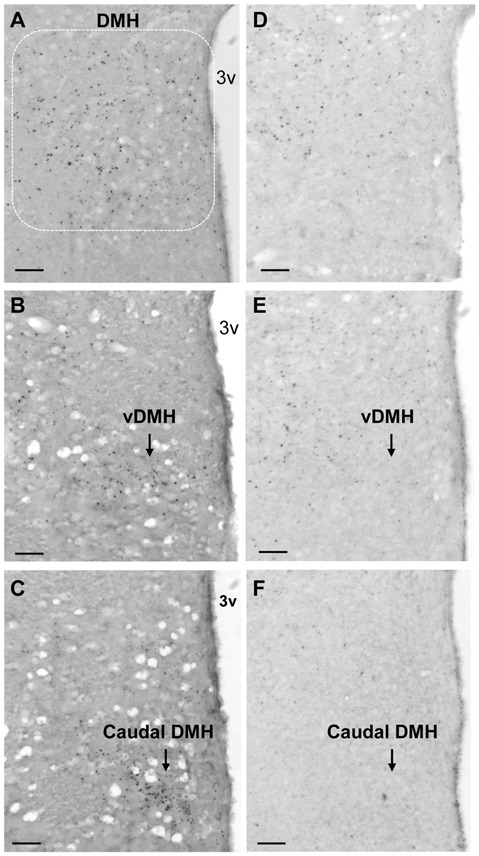
Running activity-induced c-Fos activation in the DMH. **(A–C)** Representative micrograph shows running activity-induced c-Fos activation in the DMH **(A)** particularly in the ventral subregion (vDMH, **B**) and the caudal DMH **(C)** in LEAN-RW rats. **(D–F)** c-Fos activation was decreased in the DMH **(D)** particularly in the ventral subregion **(E)** and the caudal DMH **(F)** in ZF-RW rats. Scale bar, 150 μm.

To explore brain regions that may contribute to impairments in exercise-induced reductions of food intake and body weight in obese animals with leptin signaling deficiency, we examined exercise-induced c-Fos activation throughout the entire brain in ZF rats with mutant leptin receptors using c-Fos immunohistochemistry. Lean rats with running wheel access run 480 ± 118 revolutions in 3.5 h. This running activity produced c-Fos activation in both the hypothalamus and the extra-hypothalamic areas (Figure 3A). The number of c-Fos positive cells was significantly increased in the PVN, DMH, CeA, AP and NTS (*P* < 0.05), but not in the VMH, ARC and LH in LEAN-RW rats compared to LEAN-SED rats (Figure 3A). In contrast, ZF rats with running wheel access were less active and they run 292 ± 106 revolutions in 3.5 h; about 61% of the running activity of LEAN-RW rats. As a result, running wheel access induced significant c-Fos activation in the CeA, AP and NTS in ZF-RW rats as seen in LEAN-RW rats (*P* < 0.05, Figure 3B). However, exercise-induced elevation of c-Fos activation in the PVN of LEAN-RW rats was not observed in ZF-RW rats (Figure 3B). There was a 39% increase in the number of c-Fos positive cells in the DMH of ZF-RW rats compared to ZF-SED rats, but this increase did not reach a statistical significance (*P* = 0.184, Figure 3B). Strikingly, within the DMH, exercise-induced c-Fos activation was widely distributed in LEAN-RW rats (Figure 4A), remarkably in the ventral subregion and the caudal DMH (Figures 4B,C), whereas the number of c-Fos positive cells was significantly reduced in the DMH of ZF-RW rats (Figure 4D), particularly in the ventral and caudal DMH independently of levels of running activity (Figures 4E,F). These data provide evidence suggesting that both the PVN and DMH are potential brain regions for mediating the feeding and body weight effects of physical exercise.

### Activation of DMH Neurons Promotes Running Activity and Decreases Food Intake and Body Weight

**Figure 5:**
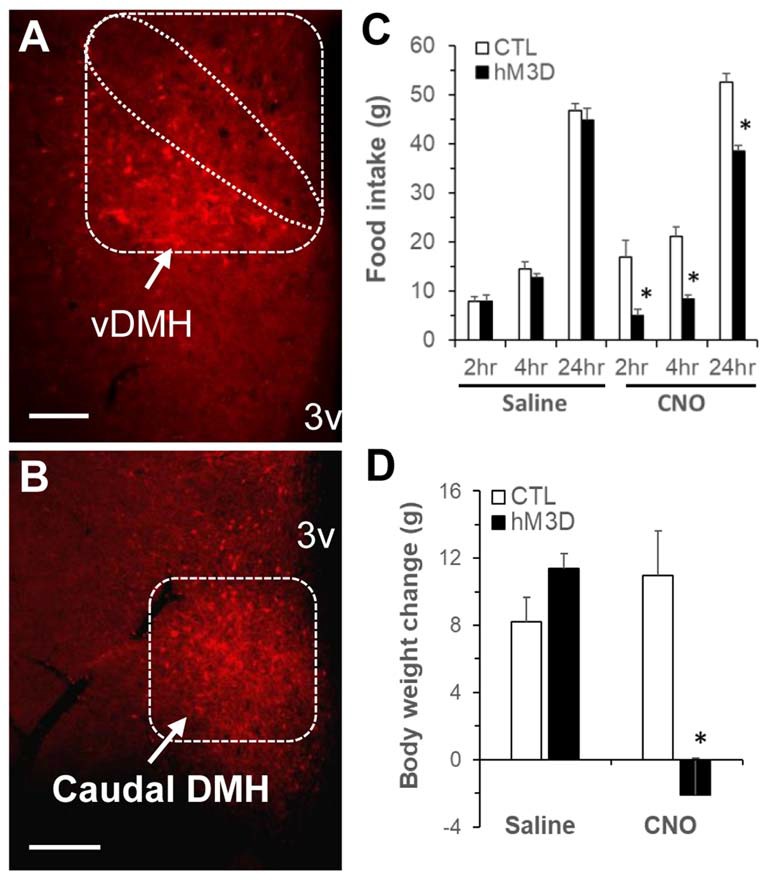
Chemogenetic stimulation of DMH neurons in ZF rats via designer receptors exclusively activated by designer drugs (DREADD). **(A,B)** Representative micrograph shows mCherry expression in the DMH post-viral DMH injection as examined under fluorescence microscopy; **(A)** the ventral subregion of the DMH (vDMH) and **(B)** the caudal DMH. **(C)** Food intake in ZF rats receiving DMH injection of the vector AAVhM3D (hM3D, *n* = 6) or the control vector (CTL, *n* = 4) at 2, 4, 24 h following intraperitoneal clozapine N-oxide (CNO; 1 mg/kg body weight, ip) or saline. **(D)** Body weight change in hM3D and CTL rats at 24 h after ip CNO or saline. Values are means ± SEM. *n* = 4–6 rats per group. *P < 0.05 vs. CTL rats. Scale bar, 250 μm.

**Figure 6:**
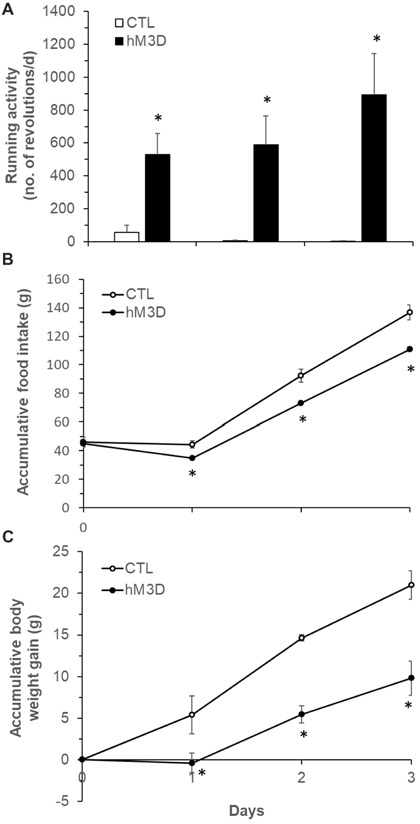
Effects of chemogenetic stimulation of DMH neurons on running activity, food intake and body weight in ZF rats. **(A)** Running wheel activity in the two groups of hM3D (*n* = 6) and CTL rats (*n* = 4) following ip CNO (1 mg/kg body weight, daily) for 3 days. **(B)** Food intake in the two groups of hM3D and CTL rats receiving ip CNO. **(C)** Body weight gain in the two groups of hM3D and CTL rats receiving ip CNO. Values are means ± SEM. *n* = 4–6 rats per group. *P < 0.05 vs. CTL rats.

Previous reports have shown that lesions of the PVN do not prevent anorectic effect of exercise (Rivest and Richard, 1990), but lesions of the DMH cause hypophagia as well as decreased running wheel activity (Bernardis, 1972). Based on these data and the present c-Fos activation results, we hypothesized that exercise-induced activation of neurons in the ventral and caudal subregions of the DMH mediates the effects of running activity on food intake and body weight. To test this hypothesis, we investigated whether chemogenetic stimulation of neurons in the ventral and caudal DMH via DREADD would affect running activity, food intake and body weight. As shown in Figures 5A,B, the AAV vector hM3D successfully infected DMH neurons (red) in the ventral subregion (Figure 5A) and its vicinity including the caudal DMH (Figure 5B) as detected with the expression marker mCherry. At an initial feeding test, running wheels were locked. There was no significant difference in food intake between CTL and hM3D rats receiving ip saline (Figure 5C), whereas ip injection of CNO resulted in significant decreases in food intake at 2, 4 and 24 h post-injection in hM3D rats compared to CTL rats (Figure 5C). As a result, while CTL rats gained 11 g of body weight in 24 h following CNO treatment, hM3D rats lost 2 g of body weight (Figure 5D). After this acute test, animals received ip CNO daily and had voluntary access to running wheels. We monitored their running activity, food intake and body weight for 3 days. As expected, CTL rats receiving ip CNO ran very few revolutions, remained hyperphagic and gained body weight rapidly (Figures 6A–C). In contrast, running activity was significantly increased in hM3D rats receiving ip CNO (Figure 6A). This treatment also resulted in significant decreases in food intake (*F*_(1,7)_ = 24.603, *P* = 0.0016, Figure 6B) and body weight gain (*F*_(1,8)_= 14.773, *P* = 0.005) in hD3M rats over 3 days compared to CTL rats (Figure 6C). Thus, these results provide support for the view that ventral and caudal DMH neurons contribute to the regulation of physical activity, food intake and body weight.

## Discussion

We have assessed a role for physical exercise in preventing obesity in ZF and SHROB rats with leptin receptor deficiency. We demonstrated that although SHROB rats had access to running wheels, the resulting physical activity did not affect their food intake and body weight. They remained hyperphagic and obese as seen in sedentary counterparts. Food restriction elevated their running activity and together these resulted in a successful reduction of body weight down to a level that was not significantly different from that of lean control rats. Consistent with leptin signaling deficiency, sedentary rats had decreased *Pomc* and increased *Npy* mRNA expression in the ARC relative to lean control rats. Regardless of this deficiency, ARC *Pomc* and *Npy* expression remained appropriately regulated in response to increased energy demands or alterations caused by food restriction and running wheel access. Levels of mRNA expression for DMH *Npy*, DMH *Crf* and PVN *Crf* were not significantly affected in rats with leptin receptor-deficiency under either sedentary or food restriction and running conditions. Intriguingly, c-Fos immunoreactivity determinations revealed that running activity induced c-Fos activation in the PVN and DMH of intact rats, but such activation (particularly in the ventral and caudal DMH) was not observed in ZF rats with leptin receptor deficiency. Chemogenetic stimulation of neurons in the ventral and caudal DMH in ZF rats resulted in significant increases in running activity and decreases in food intake and body weight. Overall, these results provide evidence that loss of exercise-induced neuronal activation in the DMH, at least in part, contributes to the ineffectiveness of voluntary running activity on hyperphagia and obesity of rats with leptin receptor deficiency, indicating a vital role for DMH neural signaling in the modulation of physical activity, food intake and body weight.

Physical exercise has long been recommended as an important non-pharmacologic intervention in preventing obesity and reducing the risk of obesity-associated comorbidities. We now acknowledge that physical exercise increases energy expenditure, leading to increases in fuel use in muscle, lipolysis in adipose tissue and fat catabolism in liver (Borer, 2003), but how food intake is modulated by exercise to affect body weight remains incompletely understood. Early study demonstrated that food intake is increased corresponding to exercise-induced increase in energy expenditure to maintain energy balance (Mayer et al., 1954). Later, growing evidence indicated that physical exercise can cause body weight loss or prevent obesity not only by increasing energy expenditure but also by reducing appetite and food intake (Levin and Dunn-Meynell, 2004; Bi et al., 2005; Irani et al., 2005; Thivel et al., 2013). For instance, voluntary running activity prevents hyperphagia and obesity of OLETF rats (Bi et al., 2005). However, physical exercise has minimal effects on obesity in animals with leptin signaling deficiency (Stern and Johnson, 1977; Dubuc et al., 1984). One explanation is that activity levels are low in these animals and such activity does not combust significant energy. The results from the present study provide compelling evidence that this ineffectiveness is because running activity did not prevent their hyperphagia. OLETF rats with access to running wheels run about 1 km/day at initial week and this running activity normalizes their hyperphagia (Bi et al., 2005). Although ad lib-fed SHROB rats had comparable running activities (1.2 km/day) to those of OLETF rats at initial week, SHROB rats remained hyperphagic and gained significant body weight as seen in sedentary counterparts. In fact, pair-fed SHROB-RW rats reduced their body weight significantly down to a level that was no longer significantly different from that of lean control rats. Thus, these results demonstrate that exercise-induced reduction of food intake is critical for the preventive effect of exercise on obesity in animals with leptin signaling deficiency.

To determine the neural mechanism underlying the ineffectiveness of exercise on food intake and body weight in these animals, we examined hypothalamic expression of *Npy*, *Pomc* and *Crf* in SHROB rats. Within the ARC, leptin acts on NPY/AgRP and POMC neurons (down-regulates *Npy/Agrp* expression and up-regulates *Pomc* expression) to decrease food intake and body weight; a loss of leptin’s actions causes increased orexigenic NPY/AgRP and decreased anorexigenic αMSH signals, leading to hyperphagia and obesity (Cone, 2006; Morton et al., 2006; Gautron and Elmquist, 2011; Friedman, 2014). Consistent with leptin signaling deficiency, sedentary SHROB rats had increased *Npy* and decreased *Pomc* mRNA expression in the ARC compared to lean control rats. Furthermore, although the body weight of pair-fed SHROB-RW rats became comparable to that of lean control rats, ARC expression of *Npy* and *Pomc* mRNA was not back to normal in pair-fed SHROB-RW rats. Levels of ARC *Npy* and *Pomc* expression were altered even greatly in pair-fed SHROB-RW rats with significantly increased *Npy* and decreased *Pomc* expression in the ARC compared to ad lib-fed sedentary SHROB rats. The pattern of changes is similar to the previous reports that voluntary exercise or food restriction results in increased *Npy* and decreased *Pomc* mRNA expression in the ARC (Zachwieja et al., 1997; Bi et al., 2003, 2005; Kawaguchi et al., 2005). Thus, in response to increased energy demands caused by food restriction and physical exercise, ARC NPY/AgRP and POMC neural systems remain appropriately regulated in SHROB rats despite leptin signaling deficiency, suggesting that ARC NPY/AgRP and POMC neural systems act to counterbalance negative energy balance resulting from food restriction and/or physical exercise. There was a caveat that the present study did not assess the regulation of ARC *Npy* and *Pomc* expression in SHROB rats in response to food restriction or running activity respectively. Thus, a specific influence of food restriction or running activity on levels of ARC *Npy* and *Pomc* expression in pair-fed SHROB-RW rats is unclear, which merits further investigation.

In addition to ARC NPY, prior work has demonstrated an important role for NPY in the DMH (particularly in the compact subregion) in the regulation of energy balance (Bi, 2013). NPY overexpression in the DMH contributes to hyperphagia and obesity of rats, whereas knockdown of DMH NPY ameliorates these alterations (Bi et al., 2001; Yang et al., 2009; Zheng et al., 2013; Kim and Bi, 2016). Importantly, voluntary exercise limits elevated expression of *Npy* in the DMH of OLETF rats (Bi et al., 2005) and acute exercise also lowers DMH NPY signaling in rats with exercise-induced anorexia (Zheng et al., 2016). In contrast to ARC NPY, DMH NPY neurons do not express leptin receptors and DMH *Npy* expression is not affected by changes of circulating leptin levels in rats (Bi et al., 2003). The present study showing no significant alterations in *Npy* mRNA expression in the DMH of sedentary SHROB rats provides additional support for the view that DMH NPY signaling is not under the control of leptin (Bi et al., 2003). Furthermore, consistent with reports that chronic food restriction and running wheel activity result in increased DMH *Npy* expression (Bi et al., 2003; Kawaguchi et al., 2005), we found a 26% non-significant increase in *Npy* mRNA expression in the DMH of pair-fed SHROB-RW rats, implying that NPY in the DMH of SHROB rats is likely regulated in response to increased energy demands as previously shown in intact rats (Bi et al., 2003; Kawaguchi et al., 2005).

Our prior work has also shown that running wheel activity induces elevation of *Crf* mRNA expression in the DMH (particularly in the dorsal subregion) in both OLETF rats and lean Sprague-Dawley rats (Bi et al., 2005; Kawaguchi et al., 2005). We have suggested that this elevation contributes to a feeding inhibitory effect of acute physical exercise (Bi et al., 2005; Kawaguchi et al., 2005). The relationship between leptin and CRF in the DMH in the control of energy balance has yet to be determined. Using mouse models, investigators have revealed an action of leptin in the dorsal subregion of the DMH in the regulation of energy expenditure and thermogenesis, but not food intake (Dodd et al., 2014; Rezai-Zadeh et al., 2014). While CRF-expressing neurons are localized to the dorsal subregion of the DMH in rats (Kawaguchi et al., 2005), leptin receptor-expressing neurons are found primarily in the ventral subregion of the DMH in rats (Bi et al., 2003). These neuroanatomical results suggest that leptin does not appear to act on DMH CRF neurons to affect physical activity in rats. The present observation of no alterations in *Crf* mRNA expression in the DMH of sedentary SHROB rats provides support for this view. In addition, in contrast to exercise-induced elevation of *Crf* mRNA expression in the DMH of lean and OLETF rats (Bi et al., 2005), *Crf* mRNA expression in the DMH was unaffected in SHROB-RW rats. The reason for this non-effect is unclear.

Lesions of the DMH result in hypophagia, hypoactivity and decreased body weight in rats (Bernardis, 1972; Bellinger and Bernardis, 2002), suggesting that while the main output of the DMH is orexigenic, the DMH (likely through other neurons) also plays an important role in the regulation of physical activity. As discussed above, NPY in the compact subregion serves as DMH orexigenic signal to modulate food intake and thermogenesis/energy expenditure (Bi et al., 2012; Bi, 2013). In contrast, the neural basis of DMH regulation of physical activity and body weight remains incompletely understood. We have found that CRF in the dorsal subregion of the DMH contributes to the effects of exercise on food intake and body weight in rats (Bi et al., 2005; Kawaguchi et al., 2005), but DMH CRF is unlikely affected by leptin. Recent mouse studies have shown that stimulation of leptin receptor-containing neurons in the dorsal subregion of the DMH promotes thermogenesis and locomotor activity to lower body weight, but does not alter food intake (Rezai-Zadeh et al., 2014), whereas activation of leptin receptor-containing neurons in the ventral subregion modulates feeding behavior (Garfield et al., 2016). In contrast to mice, leptin receptor-expressing neurons are found mainly in the ventral and caudal subregions of the DMH in rats (Elmquist et al., 1998; Bi et al., 2003), but a role for these neurons in energy balance regulation has yet to be established. In the present study, we have found exercise-induced c-Fos activation in the DMH (particularly in the ventral and caudal subregions) of intact rats, but not in ZF rats with leptin receptor deficiency. These data suggest that ZF rats have a deficit in exercise-induced activation of DMH neurons, leading to ineffectiveness of physical exercise on food intake and body weight. Based on the distribution of exercise-induced neuronal activation in the DMH as seen in rat DMH leptin expressing-neurons (Elmquist et al., 1998; Bi et al., 2003), we targeted neurons specifically in the ventral and caudal DMH in ZF rats via DREADD mediated stimulation. We found that chemogenetic stimulation of neurons in these DMH subregions of ZF rats significantly increased their running activity and decreased their food intake and body weight. Thus, these results provide evidence that ventral and caudal DMH neurons contribute to the action of the DMH in the regulation of physical activity and food intake, implying that leptin or other factors may act on these neurons to modulate physical activity, food intake and body weight.

Lesions of the PVN do not prevent the anorectic effect of exercise (Rivest and Richard, 1990), indicating that the PVN is not linked to the feeding effect of exercise. Consistent with this view, *Crf* mRNA expression is not affected in the PVN of intact rats during the period in which running activity results in significant decreases in food intake and body weight (Kawaguchi et al., 2005). We now provide evidence that *Crf* mRNA expression in the PVN was also unchanged in pair-fed SHROB-RW rats with leptin signaling deficiency. Although physical activity affects the hypothalamic-pituitary-adrenal (HPA) axis (Droste et al., 2003), in which PVN CRF acts as a positive regulator (Vale et al., 1981), a subsequent study demonstrated that activation of CRF neurons in the PVN is driven primarily by stress (Yanagita et al., 2007). While running activity did not significantly differ between the two groups of forced and spontaneous running rats, forced (stress-related) running, but not spontaneous running, resulted in significant increases in c-Fos activity in PVN CRF neurons (Yanagita et al., 2007). Our present study revealed a significant increase in running-induced c-Fos activation in the PVN in intact rats, but not in ZF rats. These results indicate that ZF rats also have a deficit in exercise-induced activation of PVN neurons, likely non-CRF neurons. The nature and function of these neurons merit further investigation.

In summary, we report that rats with leptin receptor deficiency are hyperphagic and obese, and voluntary running activity does not prevent these disorders. As expected, rats with leptin receptor deficiency have increased expression of *Npy* and decreased expression of *Pomc* in the ARC, supporting their causal roles in hyperphagia and obesity. Despite these alterations, both ARC *Npy* and *Pomc* expression remain appropriately regulated in response to food restriction and running activity. Levels of PVN *Crf*, DMH *Npy* and DMH *Crf* expression are not affected in rats with leptin receptor deficiency. However, these rats have a primary deficit in exercise-induced neuronal activation in the PVN and DMH, particularly in the ventral and caudal subregions of the DMH. Activation of neurons in these DMH subregions promotes running activity and decreases food intake and body weight. These results suggest that intact DMH neural signaling is likely crucial for the preventive effects of physical exercise on hyperphagia and obesity in obese rats lacking leptin receptors, providing potential hypothalamic targets for modulating physical activity and food intake to combat obesity.

## Data Availability

The raw data supporting the conclusions of this manuscript are available on request.

## Author Contributions

NZ designed, performed the experiments and data analysis and wrote the manuscript. LY designed, performed the experiments in SHROB rats and wrote the manuscript. LG contributed to the study design. SB directed the project, designed and supervised the study and wrote the manuscript.

## Conflict of Interest Statement

The authors declare that the research was conducted in the absence of any commercial or financial relationships that could be construed as a potential conflict of interest.
